# Risk factors for co-infections of helicobacter pylori and intestinal parasites: a cross-sectional study

**DOI:** 10.3205/dgkh000631

**Published:** 2026-02-25

**Authors:** Pourya Mohammadi, Keyghobad Ghadiri, Mosayeb Rostamian, Fereshteh Angazbany, Shahab Rezaeian, Tara Mazaheri, Tahereh Amiri, Peyman Hatami, Roya Chegene Lorestani

**Affiliations:** 1 Qazvin University of Medical Sciences, Gazvin, Iran; 2 Infectious Diseases Research Center, Health Policy and Promotion Institute, Kermanshah University of Medical Sciences, Kermanshah, Iran; 3 Tehran University of Medical Sciences, Tehran, Iran; 4 Clinical Research Development Center, Imam reza Hospital, Kermanshah University of Medical Sciences, Kermanshah, Iran

**Keywords:** H. pylori, age dependency, homemaker dependency, vegetarian diet dependency, drinking water dependency, co-infection intestinal parasites, urban area dependency

## Abstract

**Background and objective:**

*Helicobacter (H.) pylori* is one of the most prevalent bacterial infections worldwide, alongside intestinal parasitic infections, both posing significant public health risks. This research investigates the frequency of co-infections involving *H. pylori* and intestinal parasites while determining associated impacts.

**Methods:**

In this cross-sectional study, 240 patients from Imam Reza Clinic in Kermanshah (Dec 2023–Apr 2024) were included. Stool samples were tested for *H. pylori* by ELISA and intestinal parasites by microscopy and staining. Demographic data, diet, water source, and gastrointestinal symptoms were collected via questionnaire. Data were analyzed using chi-square and logistic regression in SPSS 21.

**Results:**

Of 240 patients, 43.3% had *H. pylori* infection, and intestinal parasites were detected in 24.2%. Occupation, age, vegetarian diet, and drinking water source were significantly associated with *H. pylori* infection alone. Findings suggested some parasites may increase *H. pylori* risk; however, this was not statistically confirmed. Logistic regression identified age and gastrointestinal symptoms as predictors of co-infection. Risk was highest in the 40–49 age group (OR=7.54, p=0.029); no significant associations were found in older groups (OR=4.08, p=0.148; OR=5.68, p=0.060). Multiple gastrointestinal symptoms significantly increased co-infection likelihood (OR=6.18, p=0.008). Gender (OR=1.65, p=0.267), diet (OR=1.98, p=0.134), occupation (OR=0.86, p=0.783), and water source (OR=0.21, p=0.127) showed no significant results.

**Conclusion:**

Co-infections of *H. pylori* were found in 10.8% of patients. Age and gastrointestinal symptoms were key predictors, while gender, water source, occupation, and residence showed no significant influence.

## Zusammenfassung

**Einleitung:**

*H. pylori* ist neben Darmparasiteninfektionen eine der weltweit am häufigsten vorkommenden bakteriellen Infektionen, die beide ein erhebliches Risiko für die öffentliche Gesundheit darstellen. Die Studie untersucht die Häufigkeit von Koinfektionen mit *H. pylori* und Darmparasiten und ermittelt die damit verbundenen Auswirkungen.

**Methode:**

In die Querschnittsstudie wurden 240 Patienten der Imam-Reza-Klinik in Kermanshah (Dezember 2023 bis April 2024) einbezogen. Die Stuhlproben wurden mittels ELISA auf *H. pylori* und mittels Mikroskopie und Färbung auf Darmparasiten untersucht. Demografische Daten, Ernährungsgewohnheiten, Wasserquelle und gastrointestinale Symptome wurden mittels Fragebogen erhoben. Die Daten wurden mit Hilfe von Chi-Quadrat- und logistischer Regression in SPSS 21 analysiert.

**Ergebnisse:**

Von 240 Patienten hatten 43,3% eine *H. pylori*-Infektion, und bei 24,2% wurden *Darmparasiten* nachgewiesen. Die Ergebnisse deuten darauf hin, dass einige Parasiten das *H. pylori*-Risiko erhöhen könnten; das wurde jedoch statistisch nicht bestätigt. Die logistische Regression identifizierte Alter und gastrointestinale Symptome als Prädiktoren für eine Koinfektion. Das Risiko war in der Altersgruppe der 40- bis 49-Jährigen am höchsten (OR=7,54, p=0,029); in älteren Gruppen wurden keine signifikanten Zusammenhänge festgestellt (OR=4,08, p=0,148; OR=5,68, p=0,060). Mehrere gastrointestinale Symptome erhöhten die Wahrscheinlichkeit einer Koinfektion signifikant (OR=6,18, p=0,008). Geschlecht (OR=1,65, p=0,267), Ernährung (OR=1,98, p=0,134), Beruf (OR=0,86, p=0,783) und Wasserquelle (OR=0,21, p=0,127) zeigten keinen Zusammenhang.

## Introduction

The Gram-negative bacterium *Helicobacter (H.) pylori* establishes itself within the human stomach as the primary persistent bacterial infection affecting patients worldwide. The prevalence of *H. pylori* infection ranges from 30% to 50% in developed countries, while developing countries report rates between 85% and 95% [1], [2], [3]. The pathogenic nature of *H. pylori* contributes to human gastritis and duodenal ulcers, which require extended healing time. The World Health Organization (WHO) has classified *H. pylori* as an agent that causes gastric adenocarcinoma [4]. The high prevalence of *H. pylori* infection is attributed to multiple risk factors, including poverty, poor-quality drinking water, and the presence of infected parents, as well as nationality [5], [6], [7], [8], [9]. Food contamination and transmission through the fecal-oral route serve as the primary modes of transmission. *H. pylori* infection has also been identified in cats, cockroaches, and sheep [10].

The primary group of intestinal parasites responsible for digestive infections consists of protozoa that lead to gastrointestinal symptoms [11], [12]. Worldwide, intestinal protozoan infections pose significant public health concerns, affecting approximately 3.5 billion people, particularly in developing countries [13]. The most prevalent intestinal protozoa associated with infections are *Giardia duodenalis*, *Cryptosporidium parvum*, and *Entamoeba histolytica*; however, the infection spectrum also includes *Cryptosporidium hominis*, *Blastocystis*, *Cyclospora cayetanensis*, and *Cystoisospora belli* [14], [15]. The pathogenicity of intestinal protozoan infections results in several symptoms, including diarrhea, nausea, abdominal cramps, gas production, and the appearance of fatty stools, as well as an increased risk of dehydration [16]. 

The main routes of transmission of intestinal protozoa to humans occur when infectious cysts or trophozoites are excreted in the feces of infected people or animals and are subsequently ingested by another host through contaminated water, food, hands, or objects, as well as through direct person-to-person or animal-to-person contact [15].

Microorganisms engage in various physical contact interactions with one another. Organisms involved in synergistic relationships contribute to essential ecological processes through biophysical-chemical exchanges, metabolite signaling conversions, chemotactic processes, and genetic exchanges that influence genotype selection [17], [18], [19].

This study investigates the prevalence of co-infections between *H. pylori* and intestinal parasites, while also identifying their associated risk factors.

## Material and methods

### Study design

This cross-sectional study was conducted at Imam Reza Clinic from December 2023 to April 2024 in Kermanshah, west Iran. Medical researchers collected stool samples from 240 patients suspected of harboring *H. pylori* or intestinal parasite infections. Along with the stool samples, information regarding the patient's medical history, water supply, gastrointestinal disease history, place of residence, diet, and occupational activities was also gathered.

### Inclusion and exclusion criteria

The study included adult patients presenting with gastrointestinal symptoms such as abdominal pain, diarrhea, constipation, nausea, vomiting, or bloating, who were screened for intestinal parasites and *H. pylori*.

Participants were excluded if they had a history of using anti-helminthic or anti-protozoal medications within two weeks before data collection, as these drugs could interfere with diagnostic accuracy. Additionally, patients with incomplete clinical data and those with severe comorbidities affecting gastrointestinal health were excluded.

### Formol-ether concentration technique

For parasitological examination of the samples, approximately 2 g of stool is placed in a disposable cup and thoroughly mixed with 10 mL of 10% aqueous formalin (prepared from 37% stock solution). The mixture is then passed through a cloth strainer and collected in a conical test tube. Subsequently, 3 mL of ethyl acetate is added and mixed thoroughly. The mixture is centrifuged at 2,000 rpm for 5 minutes, and the supernatant layers are discarded. A smear is then prepared from the sediment using Lugol’s iodine, and a microscopic examination is performed at 400× magnification to identify intestinal protozoa.

### H. pylori stool antigen test

To detect *H. pylori* in stool samples, an ELISA test was performed using the *H. pylori* Ag Stool kit (Pishtaz Teb) for the extraction and identification of the bacterial antigen.

### Statistical analysis

The statistical analysis was conducted using SPSS version 21. Frequency and percentage were utilized to present descriptive statistics for categorical data. The Chi-square test (χ^2^) and Fisher’s exact test were employed to analyze the relationship between *H. pylori* and co-infections with intestinal parasites, along with demographic, clinical, and lifestyle factors.

The analysis employed binary logistic regression to identify risk factors that may influence *H. pylori* infection, intestinal parasitic infections, and their co-infections. Each independent variable was assessed using the crude odds ratio (COR) along with its corresponding 95% confidence interval (CI). The study established a threshold of <0.05 for statistical significance.

### Ethics approval and agreement to participate

The protocols of the present study proposal was reviewed and approved by the Ethics Committee of Kermanshah University of Medical Sciences (IR.KUMS.REC.1403.031).

## Results

### Socio-demographic findings

During the 5-month study period, out of 240 patients, 90 (37.7%) were male and 150 (62.3%) were female. Participants were distributed across various age groups, with the highest proportions in the 30–39 years (19%) and over 60 years (18%) categories. Regarding gastrointestinal symptoms, 68 (28.3%) were asymptomatic, 59 (24.7%) experienced a single symptom, and 113 (47%) had multiple symptoms. In terms of dietary habits, 92 patients (38.3%) primarily consumed vegetables, 49 (20.4%) consumed fast food, and 99 (41.3%) had no specific dietary pattern. Occupational distribution revealed that 44 (18.3%) were self-employed, 118 (49.2%) were homemakers, and 32 (13.3%) were office workers. The majority of participants (98.8%) used tap water, while only 1.2% relied on well water. Additionally, most patients (99.6%) resided in urban areas, with only 0.4% living in rural regions.

### Distribution of H. pylori and intestinal parasitic infections

Among the 240 participants, *H. pylori* was identified in 104 cases (43.3%), while intestinal parasites were detected in 58 cases (24.2%).

**Figure 1:**
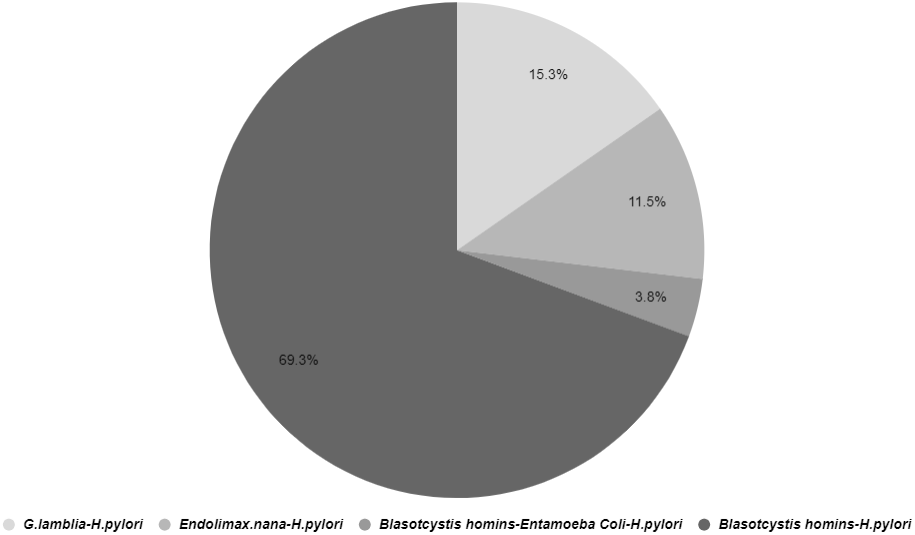
The occurrence and diversity of intestinal parasites along with co-infections involving *H. pylori*

This study revealed that co-infections of *H. pylori* and intestinal parasites were present in 10.8% of participants (Table 1 (Tab. 1)). The distribution of *H. pylori* co-infection with various intestinal parasites is illustrated in Figure 1 (Fig. 1). Co-infection with Blastocystis and *H. pylori* was observed in 69.3% of cases, making it the most prevalent combination among the study participants (Figure 1 (Fig. 1)).

### Associated factors of H. pylori and intestinal parasite co-infection

To evaluate the association between *H. pylori* infection and various demographic, clinical, and lifestyle factors, the Chi-square test (χ^2^) was used. The results revealed a significant association between *H. pylori* infection and age (p=0.001), with a higher prevalence among patients aged 50 years and older, indicating an increased risk with advancing age. Gastrointestinal symptoms were also strongly associated with infection (p=0.001), as the majority of infected patients (75%) reported multiple symptoms, highlighting the potential clinical impact of *H. pylori*. Dietary habits played a significant role (p=0.020), with higher infection rates among those who consumed vegetables, possibly indicating contamination through raw produce. Occupation was another influential factor (p=0.001), with homemakers showing the highest prevalence. Water source was significantly associated with infection (p=0.046), as all cases using well water tested positive, underscoring the potential role of contaminated water in transmission. However, no significant association was found between *H. pylori* infection and gender (p=0.420) or place of residence (p=0.252) (Table 2 (Tab. 2)).

In the analysis of intestinal parasitic infections and their association with demographic, clinical, and lifestyle factors, the findings indicated that age was a key factor linked to parasitic infections, while other variables such as gender, gastrointestinal symptoms, dietary habits, occupation, water source, and place of residence showed no statistically significant associations (Table 2 (Tab. 2)).

The Chi-square test (χ^2^) established a significant association between co-infections of *H. pylori* and intestinal parasites and the presence of gastrointestinal symptoms (p=0.001), as 77.80% of patients with co-infections exhibited multiple gastrointestinal symptoms. The analysis identified place of residence as a significant factor (p=0.004), with nearly all patients with co-infections living in urban areas. However, the analysis found no statistically significant relationships between co-infections of *H. pylori* and intestinal parasites with other variables, including gender, age, dietary habits, occupation, and water source (Table 2 (Tab. 2)).

The logistic regression identified age, gastrointestinal symptoms, and dietary habits as the primary factors determining *H. pylori* infection. Research indicates that the infection rates of *H. pylori* increased significantly among patients aged 50 to 59 years (OR=17.48, p=0.000) and those aged 60 years or older (OR=9.80, p=0.000), likely due to prolonged exposure. Patients experiencing multiple gastrointestinal symptoms during testing were found to be eleven times more likely to be infected with* H. pylori* compared to those without symptoms (OR=11.05, p=0.000). The results also indicated that vegetable consumption (OR=1.89, p=0.028) is a significant dietary factor associated with infection, as contaminated raw vegetables may contribute to transmission. Furthermore, the analysis revealed that gender, water source, occupation, and place of residence did not show any statistically significant associations with the results (p>0.05) (Table 3 (Tab. 3)).

The analysis also indicated that age was the only significant factor associated with an increased risk of intestinal parasitic infections. Patients aged 40 to 49 years had a 3.11 times higher likelihood (p=0.040) of infection compared to those in the reference group (ages 1 to 19 years). The associations for the 30 to 39 years (OR=2.76, p=0.054) and ≥60 years (OR=2.76, p=0.057) groups, while showing higher odds, were not statistically significant. Furthermore, the analysis revealed that gender, gastrointestinal symptoms, dietary habits, occupation, water source, and place of residence did not exhibit significant relationships (p>0.05) (see Table 3 (Tab. 3)).

The logistic regression also confirmed that gastrointestinal symptoms and age are major determinants leading to co-infections of *H. pylori* and intestinal parasites. Patients aged 40 to 49 years exhibited the highest susceptibility (OR=7.54) to these co-infections (p=0.029). The presence of multiple gastrointestinal symptoms increased the likelihood of co-infections between *H. pylori* and intestinal parasites by a factor of 6.18 (p=0.008) in affected patients. The associations between co-infections and other factors, such as gender (OR=1.65, p=0.267), dietary habits (OR=1.98, p=0.134), occupation (OR=0.86, p=0.783), and water source (OR=0.21, p=0.127), did not demonstrate any statistically significant relationships (see Table 3 (Tab. 3)).

## Discussion

*H. pylori *infections, when combined with intestinal parasite infections, significantly impact the gastrointestinal tract, particularly in developing countries. The high prevalence of these infections is largely attributed to poor economic conditions, unhygienic practices, and limited access to clean water [20], [21]. *H. pylori*, a common bacterial pathogen, along with intestinal parasites, serves as a major agent of gastrointestinal infections that jeopardize the health of low-income communities [22], [23]. Further research is essential, as the presence of multiple pathogens in patients leads to complex health issues that are often more severe than those caused by each pathogen acting independently.

The research found that *H. pylori* was present in 43.3% of participants, while intestinal parasitic infections affected 24.2% of participants, with Blastocystis being the most prevalent parasite at 21.25%. A study by Aklilu et al. [21] in Ethiopia reported a similar *H. pylori* prevalence rate of 14%, and another study in northeastern Ethiopia indicated an infection rate of 38.3% among participants [24]. Research conducted in China (46.6%) and Uganda (44.3%) identified *H. pylori* infection at higher levels than those observed in this study [25]. In Mexico, intestinal parasitic infections impacted 48.4% of the population, with *E. histolytica/dispar* identified as the most prevalent parasite [24]. A study in Sudan found *Giardia lamblia* in 22% of patients, whereas a study in Lebanon detected this parasite in only 5.4% of the patients examined [21]. The varying levels of these infections across different regions can be attributed to distinct sanitary conditions, access to clean water and sewage systems, as well as socioeconomic factors [21], [24].

Several research studies demonstrate that *H. pylori* and intestinal parasites exist as separate infections based on their reported findings. The research revealed a co-infection frequency of 10.8%, while studies conducted in Egypt found that Blastocystis was present in 27% of *H. pylori*-positive patients [26]. The study by Elbagi et al. indicated that gastrointestinal parasites affected 23% of patients who also had *H. pylori* infections [27]. According to Hameed et al. [25], the prevalence of multiple parasitic infections among *H. pylori*-positive patients reached 61.9%, indicating significant parasitic contamination within this population. The divergent results can be attributed to various factors, including research methodologies, participant numbers, and diagnostic procedures. Poor sanitary conditions, along with food and water contamination in countries with a low Human Development Index (HDI), such as those in Africa and Asia, contribute to increased co-infection rates. The urease-producing ability of *H. pylori* creates conditions that favor the survival of intestinal parasites as they pass through the body. Researchers require additional studies to understand how these different infections influence one another and their impact on the immune response [28].

This research failed to identify any significant relationship between *H. pylori* infection and the investigated parasites. The findings align with those presented by Moreira et al. [23], which also indicated a lack of meaningful connection between *E. histolytica* infection and *H. pylori* infection. A recent study demonstrated that adults positive for *E. histolytica* exhibited a lower prevalence of *H. pylori* compared to those without this parasite, further supporting the findings of the current study [29]. Additionally, the research conducted by Seid et al. [24] revealed that patients with *H. pylori* infections had a higher incidence of *E. histolytica/dispar* infections; however, this difference was not deemed statistically significant. Zilberberg et al. [30], along with other studies, reported an independent association between *H. pylori* and giardiasis. The discrepancies in study results may be attributed to the use of varied diagnostic methods, different participant populations, and diverse sample sizes. A comprehensive understanding of these relationships and the factors involved necessitates further rigorous investigations employing appropriate study designs.

The research findings demonstrated that *H. pylori* infection exhibited significant correlations with age, gastrointestinal symptoms, dietary habits, occupation, and water source. The study revealed that age was the only significant factor contributing to intestinal parasitic infections. The most predictive variables for co-infections of *H. pylori* and intestinal parasites were age, followed by gastrointestinal symptoms, as other variables did not show statistical significance.

Research conducted by Seid et al. [24] revealed a significant association between Giardia and *H. pylori* co-infection in patients who consume river or spring water. The studies indicate that common risk factors, such as large household sizes, reliance on unclean water sources, open defecation, rural residency, and inadequate handwashing and food hygiene practices, contribute to these infections [31], [32], [33]. According to Almaw et al. [34], drinking surface water, having a family history of *H. pylori*, and poor hand hygiene practices were identified as major risk factors for concurrent *H. pylori* and intestinal parasite infections. The research data demonstrate that environmental conditions, coupled with poor sanitary practices, drive the incidence of *H. pylori* and intestinal parasitic infections. The combination of inadequate personal hygiene, contaminated water consumption, and substandard living conditions increases patients’ vulnerability to such infections.

Research data indicates that the rates of *H. pylori* infection increase significantly with age. Age serves as the primary variable influencing parasitic infections, as all age groups above 1–19 years exhibit higher infection risks. Notably, the risk of combined infections in the 40–49 age bracket is significantly greater than in other age groups. According to Fadul et al. [35], patients over the age of 66 experience the most severe infections, a finding that aligns with our research data.

Our research determined that *H. pylori* infection and intestinal parasites showed no relationship with either gender or gender distribution. Seid et al. found that male patients experienced co-infections; however, our research did not reveal this connection [24]. A medical study conducted in Egypt discovered that gender influenced the simultaneous presence of Giardia and *H. pylori* in patients [26]. Our research did not demonstrate any correlation between gender and infections, possibly due to demographic, lifestyle, or environmental conditions that differed among the subjects.

The study revealed that patients with *H. pylori* infections primarily exhibited gastrointestinal symptoms, often accompanied by evidence of co-infections. According to Rosenstock et al. [36], *H. pylori* infection can lead to a variety of gastrointestinal symptoms in patients without peptic ulcers [36]. Research conducted by Hameed et al. [25] found that patients with *H. pylori* co-infection and intestinal parasites experienced less diarrhea compared to those with parasitic infections alone. Almaw et al. [34] documented that gastrointestinal symptoms frequently occur in conjunction with *H. pylori* and intestinal parasite co-infections, particularly involving the protozoa *G. lamblia* and *E. histolytica*. These findings contrast with results from other studies. For instance, research by Pomari et al. [37] indicated that Blastocystis, in conjunction with *H. pylori*, was commonly found in patients who did not exhibit symptoms. Additionally, studies have shown that Blastocystis and *Dientamoeba fragilis* were detected more frequently in stool samples from asymptomatic patients than in those presenting with irritable bowel syndrome [38]. Multiple studies suggest that immune system responses to these organisms can produce varying effects, sometimes alleviating symptoms while at other times exacerbating inflammatory responses.

## Conclusion

The research findings indicated that *H. pylori* and intestinal parasites co-occurred in 10.8% of the examined patients. However, the study did not statistically confirm that specific intestinal parasites increased the risk of *H. pylori* infection. It was established that age, in conjunction with gastrointestinal symptoms, served as the strongest indicators of* H. pylori* and parasitic co-infections. The data also revealed that gender, water source, occupation, and place of residence did not exhibit any significant relationships with these infections. This research underscores the necessity of improving food hygiene practices, along with public education and screenings for high-risk groups, to effectively manage co-infections.

## Notes

### Authors’ ORCIDs

- Ghadiri K: https://orcid.org/0000-0003-1678-6610
- Rostamian M: https://orcid.org/0000-0002-1071-7019
- Rezaeian S: https://orcid.org/0000-0002-5094-5315
- Chegene Lorestani R: https://orcid.org/0000-0001-7139-8456
- Mohammadi P: https://orcid.org/0000-0002-4313-3255
- Mazaheri T: https://orcid.org/0009-0004-9854-5486
- Angazbany F: https://orcid.org/0009-0002-3461-2606
- Amiri T: https://orcid.org/0009-0006-2901-2322
- Hatami P: https://orcid.org/0009-0009-2040-8365

### Ethical approval 

The present study proposal were approved by the Ethics Committee of Kermanshah University of Medical Sciences (IR.KUMS.REC.1403.031).

### Funding

None. 

### Acknowledgments

Thanks to Clinical Research Development Center of Imam Reza hospital, Kermanshah University of Medical Sciences, Kermanshah, Iran.

### Competing interests

The authors declare that they have no competing interests.

## Figures and Tables

**Table 1:**
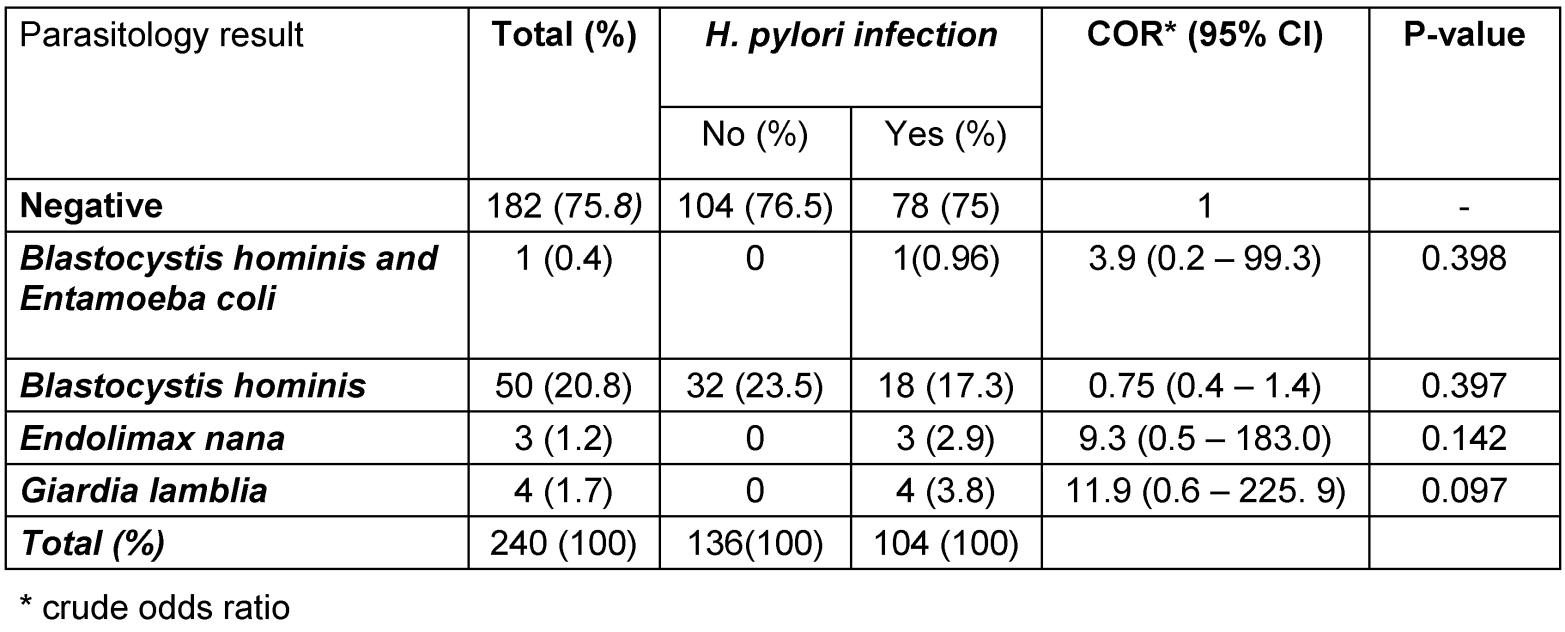
Prevalence of intestinal parasites, *H. pylori*, and coinfections among patients

**Table 2:**
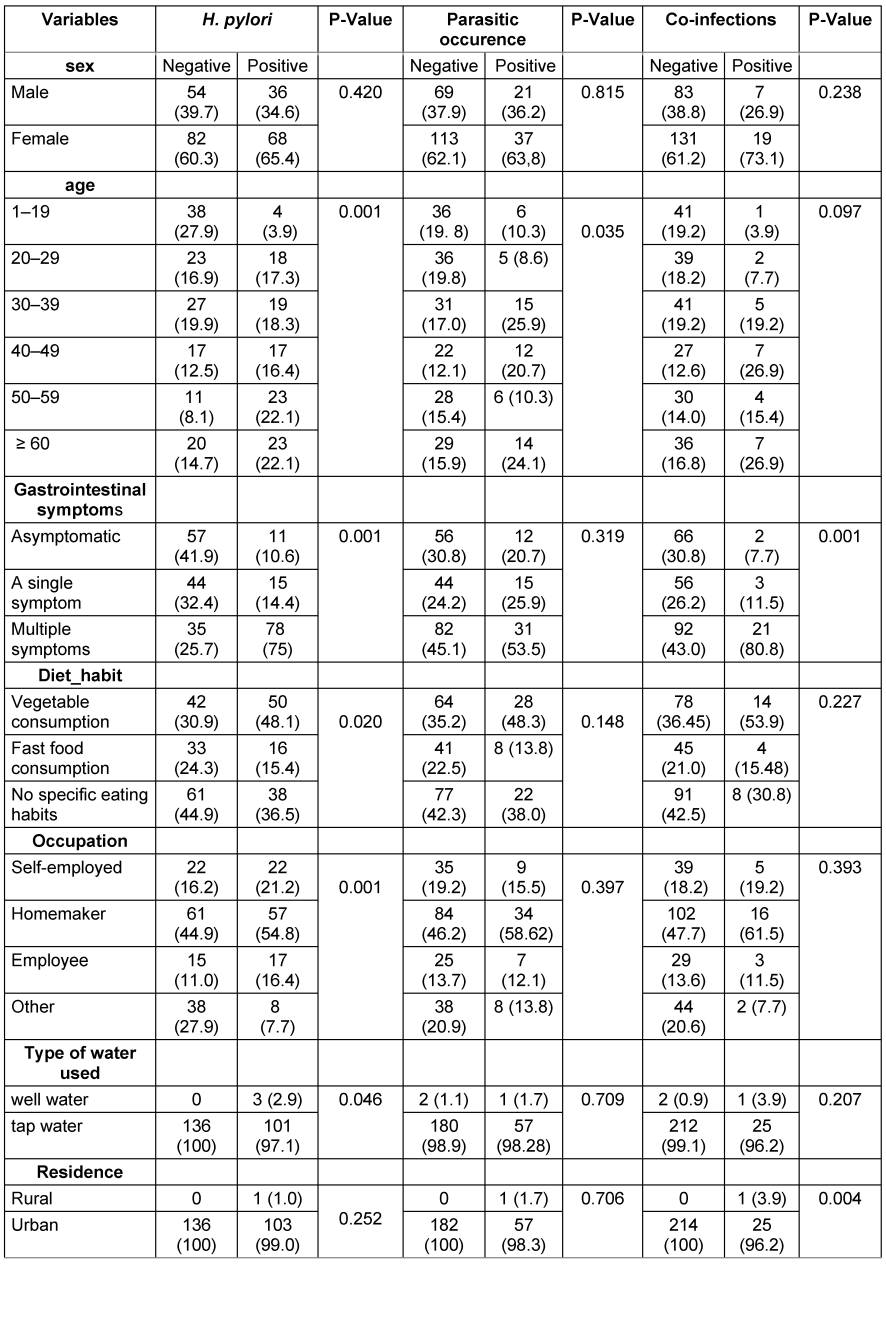
Association between intestinal parasites, *H. pylori*, and co-infections status with demographic and lifestyle factors

**Table 3:**
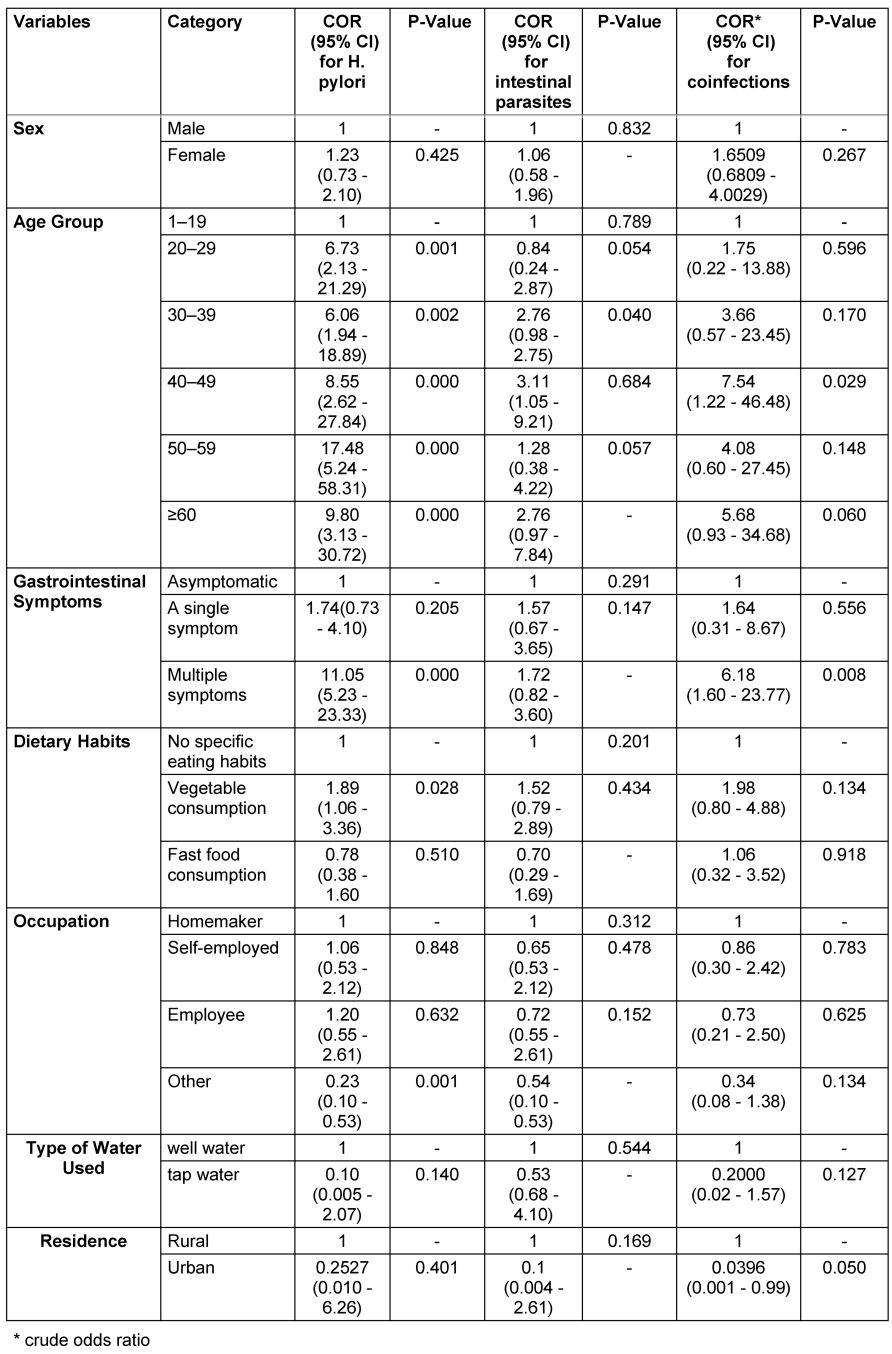
Regression analysis of factors related to *H. pylori* and intestinal parasite co-infection
